# The Pathogenesis and Treatment of Complications in Nanophthalmos

**DOI:** 10.1155/2020/6578750

**Published:** 2020-07-19

**Authors:** Ning Yang, Siyan Jin, Linlin Ma, Jia Liu, Chenli Shan, Jinsong Zhao

**Affiliations:** Department of Ophthalmology, The Second Hospital of Jilin University, Changchun City, China

## Abstract

Microphthalmos is a type of developmental disorder ophthalmopathy, which can occur isolated or combined with other ocular malformations and can occur secondary to a systemic syndrome. Nanophthalmos is one of the clinical phenotypes of microphthalmos. Due to the special and complex structure of nanophthalmic eyes, the disorder is often associated with many complications, including high hyperopia, angle-closure glaucoma, and uveal effusion syndrome. The management of these complications is challenging, and conventional therapeutic methods are often ineffective in treating them. The purpose of this paper was to review the concept of nanophthalmos and present the latest progress in the study of the pathogenesis and treatment of its complications. As it is considerably challenging for ophthalmologists to prevent or treat these nanophthalmos complications, timely diagnosis and a suitable clinical treatment plan are vital to ensure that nanophthalmos patients are treated and managed effectively.

## 1. Introduction

Nanophthalmos (NO) is one of the clinical phenotypes of microphthalmos. Due to the special anatomical structure of nanophthalmic eyes, NO is often associated with complications that can seriously damage vision and even cause blindness if they are not properly managed. The purpose of this paper was to review the concept of NO and present the latest progress in the study of the pathogenesis and treatment of the complications of NO. Previous studies have focused on the comparison of different treatment methods or the proposal of new treatment ideas. There is no comprehensive review of the mechanism and treatment of the complications of NO. This paper reviews the available literature on the complications of NO and their prevention and treatment in the past 20 years, with a greater focus on reports published in the last five years.

## 2. Microphthalmos and Nanophthalmos

Microphthalmos (MO) is a developmental ocular disorder [1] and is characterized by eyeballs with ocular axial lengths at least two standard deviations smaller than the average axial length of eyes in normal control age groups [2]. A European study reported that the prevalence of MO is between 0.002% and 0.017% in the European population [3]. MO is divided into simple MO and complex MO dependent on whether other eye deformities or systemic diseases are present. The simple MO is a small eyeball with normal shape, except for the short axial length; it does not incorporate other eye deformities or other systemic diseases [4]. The complex MO occur alongside other ocular malformations, including iris coloboma, chorioretinal coloboma, persistent fetal vasculature, and retinal coloboma [5, 6]; it can also occur as part of some syndromes, including the Bosma arhinia microphthalmia syndrome and the Gorlin–Chaudhry–Moss syndrome [7].

**Figure 1:**
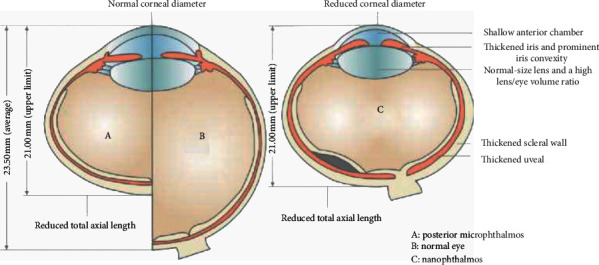
The clinical spectrum of the simple microphthalmic phenotype is nanophthalmos and posterior microphthalmos. Nanophthalmos has a short axial length due to small anterior and posterior segments with thickened choroid and sclera and normal lens volume. Posterior microphthalmos has a short axial length due to a reduced posterior segment dimension with normal anterior chamber dimensions.

Nanophthalmos (NO) is one of the clinical phenotypes of simple MO; anterior and posterior segments of the eyeball do not develop into a normal size (Fig.  1) [8]. NO is a result of developmental arrest in the eyeball after the closure of the embryonic fissure and usually affects both eyes [9]. NO can manifest as sporadic or familial disease, autosomal dominant or recessive inheritance, and the current study found that five genes (MFRP, TMEM98, PRSS56, CRB1, and BEST1) are related to the family form of the NO eye [10–14]. Mutation of the MFRP gene is believed to alter axial globe growth through a Wnt signalling mechanism [15]. The physiologic and pathogenic mechanisms of other four genes remain to be fully determined.

There is no consensus on the diagnostic standard of NO. Wu et al. [16] reported that the conditions for diagnosing NO eyeballs include an axial length <21 mm, posterior scleral wall thickness >1.7 mm, corneal diameter <11 mm, anterior chamber structure crowded, and high hyperopia. Yalvac et al. [17] used the characteristics above, but they define the axial length as <20.5 mm and increase the lens/eyeball volume ratio as one of the diagnostic conditions. In addition, some patients with NO can be observed with increased choroid thickness, macular folds, and the anomalies of the foveal avascular zone [18–20]. High hyperopia is usually seen as the first symptom of NO in childhood and is often accompanied by complications such as angle-closure glaucoma (ACG) and uveal effusion syndrome (UES) in adulthood, which further reduces vision [21]. It should be noted that NO needs to be distinguished from posterior microphthalmos (PM), another clinical phenotype of simple MO, which manifests as abnormal development of the back of the eyeball with a normal corneal diameter, anterior chamber depth, and lens/eyeball volume ratio (Fig.  1) [22]. Relhan et al. [21] found that the incidence of angle-closure glaucoma was 69.2% and 0% in the NO and PM groups, respectively, which suggests a huge difference in the risk of complications between NO and PM. Therefore, close follow-up should be scheduled for early detection of ACG.

## 3. Complications of Nanophthalmos and Their Pathophysiological Bases

### 3.1. High Hyperopia

High hyperopia is a characteristic clinical manifestation of NO and occurs mainly due to the short axial length and increased lens/eye volume ratio of nanophthalmic eyes, which causes objects to be imaged behind the retina [4]. NO patients are often diagnosed in the middle and late stages of life because of secondary complications such as ACG and UES and are missed in the early stages of life because they only show high hyperopia, which can be corrected after wearing glasses [21]. Due to the characteristic high hyperopia that is seen in NO, the patients usually require an intraocular lens (IOL) implant with a refractive power ranging from +45 D to +60 D, a power range that presents a challenge in terms of choice of lens types and implantation methods [23, 24].

### 3.2. Angle-Closure Glaucoma

The nanophthalmic eye has a short axial length and a shallow anterior chamber, but the size of the lens is normal [25]. This results in an increase in the lens/eyeball volume ratio from 4% under normal conditions to 10–30% at the pathological level [17, 26, 27]. ACG is often present in NO patients between the ages of 40 and 60 years, which may be related to the thickening of the lens with age [28]. The mechanism of angle closure may be multifactorial. The large lens pushes the iris towards the crowded anterior chamber in NO, which leads to pupillary block and eventually causes the formation of peripheral anterior synechia (PAS) [17]. The thickened and structurally abnormal sclera in nanophthalmic eyes may cause annular ciliochoroidal effusion or ciliary body detachment, which leads to physical displacement of the peripheral iris and eventually causes the anterior chamber angle to close [25]. In addition, uveal effusion allows the lens to move forward, by relaxing the ciliary zonules and increasing the contact of the iris lens, leading to a pupillary block [28–30].

### 3.3. Uveal Effusion Syndrome

UES is a very rare disease characterized by choroidal effusion with serous choroid and/or retinal detachment [31, 32]. It manifests as a visual field defect in its early stage; severe vision loss occurs when the retinal detachment involves macular or secondary retinal pigment epithelial degeneration [32]. It is an exclusive diagnosis that is made when the cause of uveal leakage cannot be determined, which is usually in cases of eyes with normal intraocular pressure (IOP) values and without significant inflammation [32]. Uyama et al. [33] divided UES into three types according to the case pathology: eyes with type I UES, which is the most common type of UES, are characterized by a short axial length and an abnormal sclera (as is seen in NO); eyes with type II UES are characterized by the scleral thickening but a normal axial length; and eyes with type III UES, also known as idiopathic uveal leakage, have normal axial lengths and sclerae. The current opinion is that the abnormality of the sclera of NO, including scleral thickening and disorganized collagen fibres of the sclera, results in compression of the vortex veins and reduced permeability of scleral macromolecular substances, thereby leading to UES [32].

Regarding compression of the vortex vein, disorganized collagen fibres may lead to compression of vortexes, congestion of the choroidal vein, and uveal effusion [18, 19]. In pathological states, such as in cases of iridocyclitis or detachment of the ciliary body and choroid, the impact of this mechanism will further increase [28]. The treatment of uveal effusion in nanophthalmic eyes by vortex vein decompression suggests that the compression of the vortexes is one of the causes of these complications [29].

Regarding decreased scleral protein permeability, Gass and Ward et al. [34, 35] believed that the disordered collagen fibres in the sclera of NO patients slow down the outflow of macromolecular substances, including proteins and glycosaminoglycans (GAG), through the sclera. The results of some studies are in line with this view; Okabe et al. [36] found that subretinal fluid from the eyes of patients with UES contained an abnormally high concentration of albumin, up to 26.5 g/dl, which is higher than that in serum. Ward et al. [34] found that the deposition of GAG-like substances in the scleral matrix of patients with UES increased, whereas Uyama et al. [33, 37] confirmed that the removal of GAGs can increase the outflow of scleral fluid. High osmotic pressure caused by the reduced outflow of these macromolecular substances will further increase fluid retention in the suprachoroidal space [35].

Under physiological conditions, the water in the subretinal space can be drawn by the dehydrating power of the pigment epithelium and the retinal epithelial physiological pump [35]. However, due to the abnormal sclera in NO eyeballs, the decomposition of the pigment epithelium caused by choroidal vascular congestion and detachment weakens the compensatory effect of the retinal pigment epithelium pump and allows water and these macromolecules to move from the suprachoroidal space to the subretinal space [33, 36, 38]. The high permeability in the subretinal cavity causes fluid retention and further retinal detachment [35, 39–41].

## 4. Treatment in High Hyperopia in Nanophthalmic Eyes

The visual impairment caused by high hyperopia in nanophthalmic eyes reduces the quality of life of patients with NO. Previous studies have suggested that refractive surgery for correcting hyperopia is not suitable for NO patients because reliable and predictable results cannot be achieved in cases of extreme refractive errors using methods including laser in situ keratomileusis (LASIK) and photorefractive keratectomy [42, 43]. In a recent study, satisfactory results were obtained after the treatment of high hyperopia (up to +7.50 D) using LASIK [44]. However, there have been no reports of using LASIK for the treatment of high hyperopia in nanophthalmic eyes. In the past, the treatment of hyperopia in nanophthalmic eyes was done using thicker spectacle lenses or contact lenses. Presently, the refractive lens exchange is considered a more suitable therapeutic choice [45]. Phacoemulsification and IOL implantation can reduce the spherical equivalent of the refractive errors of nanophthalmic eyes, thereby reducing the thickness of the glasses required to correct any residual errors and improving the quality of life of the patients [24]. Currently, there are multiple IOL implantation methods to choose from. A single foldable IOL with a relatively high dioptric power (+30 D and +40 D) can be implanted; this method does not completely correct the refractive error, and the patient will still require glasses after surgery. In another method, a high-refractive polymethyl methacrylate (PMMA) IOL, which can provide up to +75 D diopters of correction, can be implanted [46]. However, PMMA IOLs are harder and cannot be folded; therefore, the surgical incision used in this method is larger, increasing the risk of intraoperative and postoperative complications [46]. Piggyback IOL implantation (one IOL in the capsular bag and the other in the ciliary sulcus) is another method that can be used for the treatment of high hyperopia in nanophthalmic eyes. This method can provide dioptric correction above +40 D, but it can cause anterior segment-related complications, including iris pigment loss, iris inflammation, and peripheral anterior and posterior adhesions [42, 47]. New IOL implantation methods have been tried in recent years. The 2015 report by Singh et al. [48] showed that implantable foldable aspherical single focus IOLs in nanophthalmic eyes can significantly improve uncorrected distance refractive error and reduce its spherical equivalent after surgery. However, the improvement of distance vision after correction was not obvious. A foldable aspherical single focus IOL has a refractive power between +45 D and +60 D and can be implanted through a 2.2 mm incision. The small incision size used for implanting this IOL eliminates the problems caused by the large incisions used for implanting PMMA IOLs and reduces the risk of anterior segment-related complications caused by piggybacking IOLs [48]. On another note, recent studies have shown that mutations in the human membrane-type frizzled-related protein gene can cause NO. Gene therapy can normalize the axial lengths of nanophthalmic eyes and reverse their high hyperopia, but gene therapy studies in this regard are still in the animal research stage [49].

## 5. Treatment of Angle-Closure Glaucoma in Nanophthalmic Eyes

It is difficult to treat NO patients with ACG in the clinic, especially if they have persistent high IOP that cannot be controlled with drug and laser therapy and require surgery. In addition, NO patients are often prone to serious complications, such as expulsive haemorrhage, persistent shallow anterior chamber, and aqueous misdirection syndrome, during or after intraocular surgery [50–52].

### 5.1. Drugs and Laser Therapy

Patients with NO are at a higher risk of having intraoperative and postoperative complications; therefore, for patients who do not have angle adhesion or whose angle adhesions are not extensive, drug and laser treatments should be administered as early as possible [41]. Commonly used drugs include hypertonic agents and *β*-blockers. Hypertonic agents can condense the vitreous body and provide space for the lens to move backwards to relieve pupillary block, and *β*-blockers can regulate IOP by reducing the secretion of the aqueous. It should be noted that miotics should be used with caution in nanophthalmic eyes because the relaxation of the suspensory ligament and the secondary forward movement of the lens will cause the anterior chamber to become shallower. Generally, although drug therapy is the first step of treatment, it is not very effective for NO patients. Laser iridotomy is recommended to relieve pupillary block in cases of ACG secondary to NO [28, 53], but sometimes iridotomy is not enough [54]. Laser peripheral iridoplasty can be considered if the anterior chamber angle remains closed after laser iridotomy [9, 28]. However, laser iridotomy and laser peripheral iridoplasty are effective in the early stage of the disorder, but they may not adequately regulate IOP because of the occurrence of peripheral anterior synechiae.

### 5.2. Surgical Treatment

If IOP cannot be controlled after drug therapy combined with laser treatment, glaucoma filtering surgery should be considered, although serious complications often occur during or after the surgery. Singh et al. [53] reported that IOP control was not achieved for 60% of 15 NO patients who underwent glaucoma filtration surgery; 86.6% of the patients suffered from continuous visual loss. Therefore, it is necessary to find an alternative method of filtering surgery or combining other surgical methods with filtering surgery to reduce the incidence of intraoperative complications and increase the success rate.

One of such options is combination with sclerectomy, punch sclerostomy, and vortex decompression. Intraocular surgery in NO is usually accompanied by some intraoperative and postoperative complications, such as uveal effusion, which is considered a serious threat to vision [17, 27, 55, 56]. The sclera of a nanophthalmic eye is usually thick, and the thickened sclera may compress the vortex vein and reduce the permeability of the sclera. A sudden decrease in intraocular pressure during surgery may trigger the rapid development of uveal effusion [27]. Sclerectomy and vortex decompression during surgery can relieve this compression, and punch sclerostomy can drain choroidal effusion, but complications such as uveal effusion can still occur in some nanophthalmic patients after these interventions [17, 55, 57, 58].

A second option is a combination of vitrectomy, lensectomy, and IOL implantation. The disproportionate lens plays a key role in the cause of pupillary block in NO eyes [25]. The removal of the lens can reduce the contents of the eyeball to deepen the anterior chamber, which can eliminate pupillary block and establish aqueous outflow reconstruction [59]. In addition, the excision of the anterior vitreous can create enough space for the iris lens diaphragm to move backwards, which can solve the adhesion of the vitreous to the iris or lens [27, 56]. Therefore, a combination of vitrectomy and lensectomy is considered a suitable method to solve this complicated situation. The anterior chamber of the nanophthalmic eye is very shallow and often has corneal oedema, so phacoemulsification is not considered a treatment option [27, 60]. The use of mechanical cutting, instead of ultrasound energy, in crystal removal can minimize the damage to endothelial cells. Zhang et al. [27] reported that after 21 patients with NO and ACG were treated with a vitrectomy combined with a transscleral lens extraction, the pupil block was eliminated, and the intraocular pressure was significantly reduced. It was found that the incidence of postoperative uveal effusion is low, which may be due to intravitreal lavage; this can provide stable and adjustable IOP control and avoid rapid fluctuations in the IOP throughout the surgery [27, 60, 61].

Simultaneous implantation of an IOL can improve interventional vision as well. However, IOL implantation poses a higher risk for NO patients because of the complicated condition of their eyes; besides, the postoperative vision improvement in NO cases is not obvious. Considering this result, Zhang et al. [27] do not recommend IOL implantation. However, Sharan et al. [62] put forward a different point of view; they used the new generation anterior segment diagnostic equipment, Pentacam, and ultrasound biomicroscopy to observe the anterior chamber angle of NO patients with refractory ACG and found that the anterior chamber depth of the patients increased, and their anterior chamber angle was open after IOL implantation, but the number of such observations was small. Further research is needed to ascertain whether IOL implantation for patients with NO can prevent the development of trabecular meshwork dysfunction, which in turn prevents the occurrence of ACG and optic neuropathy. Studies are also required to determine who can benefit from early intervention.

In summary, drug and laser treatment may be effective before PAS is formed for nanophthalmic patients with ACG; surgical treatment is regarded as the last choice treatment method if these measures are not effective. B-ultrasound and ultrasound biomicroscopy examinations are necessary before surgery [28, 56]. If there is choroidal thickening or any suspicion of uveal effusion during preoperative examination, partial quadrant sclerectomy or sclerectomy should be performed before intraocular surgery to minimize the risk of uveal effusion during and after surgery. If preoperative examination excludes the possibility of uveal effusion, a single lensectomy combined with pars plana vitrectomy surgery is safe and effective, and the incidence of intraoperative uveal effusion is low. In addition, a 100 ml intravenous infusion of 20% mannitol before starting the procedure can shrink the vitreous and minimize positive vitreous pressure, which better controls the IOP [28]. General anaesthesia surgery helps stabilize the patient's mood and blood pressure and to some extent reduces IOP fluctuations [56].

## 6. Treatments for Uveal Effusion Syndrome in Nanophthalmic Eyes

### 6.1. Surgical Treatment

The pathogenesis of UES in NO is primarily scleral thickening, which causes uveal effusion by obstructing protein diffusion out of sclera and obstruction of vortex veins [32]. Vortex vein decompression is the first surgical method for treating UES and can directly decompress the vortex veins, but the separation of the vortex vein has been rarely used in recent times because of its technical difficulties and the high risk of vein rupture [31]. In addition to the direct effect of choroid vein decompression on choroidal vessels, simply performing a sclerotomy and/or sclerectomy can decompress vortex veins indirectly by relaxing the scleral tension or reducing resistance to the outflow of large molecules [32, 35]. Some studies have reported on different surgical methods and their postoperative effects in recent years. Some of those methods include the following:

1. Partial-thickness sclerectomy (PTS): Ozgonul et al. [63] recommends using graded surgery for NO patients with UES. The first method is PTS (two-thirds of the scleral thickness), which has been proven effective in some patients. The resistance of the transscleral sclera to protein movement decreases after sclerectomy, although this may be affected by the amount of sclera excised and postoperative fibrous scarring [63]. A prospective study showed that partial quadrant PTS (90% of the scleral thickness) in nanophthalmic eyes with UES had a significant effect [64], and PTS produced better results in the four quadrants [65]. The authors of the study considered that the PTS performed in the four quadrants could reduce the overall resistance to subchoroidal fluid outflow, thereby promoting the outflow of fluids in the suprachoroidal and subretinal spaces [64, 65]. However, due to the large area of sclera removed, current technology may cause dilation of the sclera [64].
2. PTS combined with mitomycin C (MMC): episcleral scarring can lead to closure of scleral windows [63]. MMC is a drug used to prevent scleral scar formation after scleral surgery. Ozgonul et al. [63] reported that PTS alone is not effective for treating some NO patients with UES because of the possibility of fibrous tissue hyperplasia occurring after surgery; however, repeating the same PTS method in all four quadrants or performing PTS combined with MMC was shown to be effective. Akduman et al. [66, 67] also confirmed this conclusion.
3. PTS combined with punch sclerostomy: Uyama et al. [33] performed PTS (two-thirds of the scleral thickness) on the temporal and nasal quadrants and performed sclerostomy under the scleral flap in six eyes of NO patients with UES. Retinal detachment gradually resolved in four eyes within one to two months after surgery, and two eyes underwent additional retinal reduction in the upper quadrant after the same surgery [33]. The report from Ozgonul et al. [63] showed that although the choroidal effusion in some nanophthalmic eyes with UES did not resolve after multiple PTS procedures, the punch sclerostomy based on the previous PTS technique was an effective treatment method. Scleral ostomy can be used when the visual field is considered too poor to perform a controlled scalpel dissection, especially if the operation has been performed previously [68].
4. Full-thickness sclerotomy (FTS): Kong et al. [69] reported that single FTS without sclerostomy under the scleral flap can produce good results, can simplify the surgical procedure, and reduce the operation time. Additionally, FTS plays an immediate role by promoting the drainage of uveal effusion; FTS can also indirectly decompress the vortex vein by relaxing the tension in the sclera [69].

### 6.2. Drug Therapy

There are few studies on the use of drug therapy for the treatment of UES in nanophthalmic eyes. Guo et al. [70] defined intractable UES as a case of UES in which the choroidal leakage recurs or does not subside within four weeks after partial sclerectomy. They found that the concentrations of interleukin-(IL-) 6, IL-8, and the vascular endothelial growth factor (VEGF) in the aqueous humour of NO patients with intractable UES were higher than those of patients in a control group. Moreover, they reported the regression of subretinal fluid after anti-VEGF therapy [70]. The mechanism behind this may be that VEGF can reduce the activation of macrophages and further regulate the expression of IL-6 and IL-8 [71, 72]. The reports of Park and Andrijevic Dert et al. [73, 74] show that oral carbonic anhydrase inhibitors and local prostaglandin analogues can help the absorption of subretinal fluid and improve the detached choroid and retina in nanophthalmic eyes with UES. There may be two possible mechanisms for this: on one hand, the prostaglandin analogues decrease the level of scleral collagens by increasing the level of scleral metalloproteinase, thus regulating the flow of fluids through the sclera and increasing the permeability of scleral macromolecules [75]. It should be noted that a large sample case-control study is required to determine the safety and effectiveness of drug therapy for treating UES in nanophthalmic eyes and exclude the possibility of spontaneous remission in such cases. However, the incidence of UES in nanophthalmic eyes is so low that such a study will be difficult to implement.

In conclusion, surgery is a common method for treating UES in nanophthalmic eyes. Despite the poorly understood aetiology of nanophthalmic UES, some surgical methods based on removal of thickened sclera without vortex vein decompression is often successful. Sclerotomy is chosen by most people in the literature, and its efficacy seems to be better than partial-thickness sclerectomy. Compared to a small sclerostomy under the scleral flap, full-thickness sclerotomy could simplify the operative procedure and reduce operation time and complications, such as bleeding and hypotony. MMC should be actively applied during surgery because it can reduce the risk of episcleral scarring, which can lead to closure of scleral windows. However, reoperation is necessary when initially successful sclerectomy surgery subsequently fails due to late fibrosis and scarring. Full-thickness cuts with the scleral punch may be safer during reoperation because a rounded instrument rather than a sharp edge is presented against the choroid. Drug therapy has a certain value for patients who show unsatisfactory results after surgical treatment or have severe scleral scarring after multiple surgeries. In addition, patients who refuse surgical treatment can choose drug therapy instead.

## 7. Conclusion

This is a systematic review of the existing research on the treatment of NO complications, which will be of value to ophthalmologists. This review shows that every ophthalmologist must understand the complications of nanophthalmos and the optimal treatment methods for them. This will aid the development of individualized treatment strategies based on the conditions of each patient to ensure reduction of complications and improvement of safety and effectiveness of treatment. Although the scope of the search was extended to 20 years, we did not find a large sample case-control study on NO because of the rarity of NO. Our evaluation of the effects of each procedure suggested a general guidance for the minimizing treatment complications. In recent years, reports on the genetic diagnosis of NO, the establishment of early imaging screening methods, and the measurement of IOL powers in patients with short axial length have gradually increased. These advances should be closely researched to clarify the best treatment strategies for NO.

## 8. Method of Literature Search

A literature search was performed on the PubMed from 1999 to 2020. Search terms included “microphthalmos,” “nanophthalmos,” “complications,” “glaucoma,” “uveal effusion syndrome,” and their combinations. Further articles were identified from the reference lists of retrieved articles. Reports published only as abstracts were excluded, as they were non-English language articles.
